# High Response Rate and Prolonged Survival of Unresectable Biliary Tract Cancer Treated With a New Combination Therapy Consisting of Intraarterial Chemotherapy Plus Radiotherapy

**DOI:** 10.3389/fonc.2020.597813

**Published:** 2020-11-17

**Authors:** Takuma Goto, Hiroya Saito, Junpei Sasajima, Toru Kawamoto, Akihiro Fujinaga, Tatsuya Utsumi, Nubuyuki Yanagawa, Kazuhide Hiramatsu, Akio Takamura, Hiroki Sato, Shugo Fujibayashi, Mikihiro Fujiya

**Affiliations:** ^1^Division of Gastroenterology and Hematology/Oncology, Department of Medicine, Asahikawa Medical University, Asahikawa, Japan; ^2^Department of Gastroenterology, Asahikawa Kousei Hospital, Asahikawa, Japan; ^3^Department of Radiology, Asahikawa Kousei Hospital, Asahikawa, Japan; ^4^Department of Radiology, Sapporo Higashi Tokusyukai Hospital, Sapporo, Japan; ^5^Department of Internal Medicine, Engaru Kousei Hospital, Engaru, Japan

**Keywords:** biliary tract cancer, gallbladder cancer, radiation therapy, arterial infusion chemotherapy, chemotherapy

## Abstract

**Synopsis:**

A new combination therapy consisting of intraarterial chemotherapy plus radiotherapy was demonstrated to have the potential to improve the response rate and survival time in patients with unresectable biliary tract cancer.

**Purpose:**

We retrospectively investigated the effectiveness and safety of a new combination therapy consisting of intraarterial chemotherapy plus radiation therapy (AI+RT), which may have the potential to improve unresectable biliary tract cancer (BTC).

**Methods:**

We retrospectively reviewed 52 BTC cases treated with AI+RT and analyzed the anti-tumor effect, survival time and adverse events. The AI+RT regimen consisted of one-shot intraarterial chemotherapy (AI) at the first angiography session, almost 6 months of reservoir AI (5-FU and cisplatin, q/week) and external radiation with a maximum dose of 50.6 Gy.

**Results:**

The response rate and disease control rate were high, at 40.4% and 96.2%, respectively, and the median overall and progression-free survival time were 463 and 431 days; thus, long-term survival was achieved. A univariate analysis identified 12 prognostic factors, and a performance status of 2 (hazard ratio [HR]: 4.82, p=0.02), jaundice (HR: 3.22, p<0.01), peritoneal dissemination (HR: 22.5, p<0.01), number of AI (HR: 0.35, p=0.01) and response to AI+RT (HR: 0.23, p<0.01) were extracted as significant prognostic factors in a multivariate analysis. The following: grade ≥3 adverse events occurred: leucopenia (11.5%), neutropenia (1.9%), anemia (15.4%), thrombocytopenia (11.5%), anorexia (3.8%), gastroduodenal ulcer (25.0%), and cholangitis (23.1%). There were no cases of treatment-related death.

**Conclusions:**

AI+RT was shown to contribute to a high response rate and prolonged survival in patients with unresectable BTC. A sufficient number of AI and the response to this therapy were thought to be significant prognostic factors in patients receiving AI+RT. Advances in multidisciplinary therapies, such as AI+RT, which was described in the present study, are also considered to be important for the future.

## Introduction

The incidence of biliary tract cancer (BTC) appears to be increasing in Europe, Latin America and East Asia. In Japan, in particular, there are approximately 20,000 new cases each year, and BTC is currently the sixth leading cause of cancer-related mortality in Japan (1).

Patients with BTC have a poor prognosis, with 5-year survival rates of <10% (2, 3), so effective treatment strategies are urgently required. Surgical resection currently represents the only potentially curative treatment for BTC, but 70% of patients are deemed unresectable (4), and about half of patients undergoing resection relapse within 1 year after resection (5), so the 5-year survival rates remain low (33.1% for bile duct cancer [BDC], 52.8% for ampullary cancer and 41.6% for gallbladder cancer [GBC]) (6). Therefore, most cases require treatment to unresectable or relapse BTC.

At present, gemcitabine and cisplatin (CDDP) (GC) therapy is the primary first-line systemic chemotherapy for BTC (7, 8), while gemcitabine plus oxaliplatin (GEMOX), capecitabine plus oxaliplatin (CapeOX) and gemcitabine plus S-1 (GS) therapy have been considered as alternatives. However, all of these therapies have shown an insufficient response rate of 20% to 30%, and the median overall survival (mOS) and median progression-free survival (mPFS) remain around 1 year and 6 months respectively (9–14).

At our hospital, intra-arterial chemotherapy (AI) has been used for liver metastasis of cancer. The etoposide + epirubicin + CDDP therapy (EEP) used in one-shot AI was reported to be a highly responsive treatment for gastric cancer, but there were many adverse events, and it did not become established as a systemic chemotherapy (15). We have been using EEP with one-shot AI because of its high response rate and low number of adverse events. An improved prognosis was also confirmed when using radiation therapy (RT) combined with AI (16), and AI+RT has already been performed for other cancers at our hospital. We therefore focused on AI+RT, which have been reported only domestically as a potential treatment regimen for unresectable GBC and as a possible alternative to systemic chemotherapy.

The present study retrospectively examined the usefulness and safety of AI+RT in BTCs and clarified its potential utility in the future.

## Materials and Methods

### Patients

A single-center, retrospective evaluation was performed. This study included 52 patients with unresectable BTC who were diagnosed by computed tomography (CT), magnetic resonance imaging (MRI), endoscopic ultrasound (EUS) imaging and pathological findings, and treated by combination therapy of AI+RT as first-line therapy at Asahikawa Kosei Hospital. Patients with BTC, including GBC and BDC, were analyzed in this study. BDC included hilar cholangiocarcinoma and distal bile duct cancer but not intrahepatic cholangiocarcinoma (ICC). This therapy was performed in cases with locally advanced lesions, lymph node metastasis and liver metastasis. However, cases with distant metastases were excluded when the physicians judged the patients to be unsuitable for treatment. All 52 cases had started AI+RT by April 2011 and were followed until December 31, 2018. We used the TNM classification of malignant tumors of the International Union Against Cancer version 8 (UICC 8).

We used the opt-out approach to give subjects the opportunity to decline study participation, as it is virtually impossible to obtain informed consent for a retrospective review. This retrospective study was approved by the Medical Ethics Committee of Asahikawa Medical University (Number: 16180) and Asahikawa Kousei Hospital (Number: 2876).

### Method of Therapy

**Figure 1:**
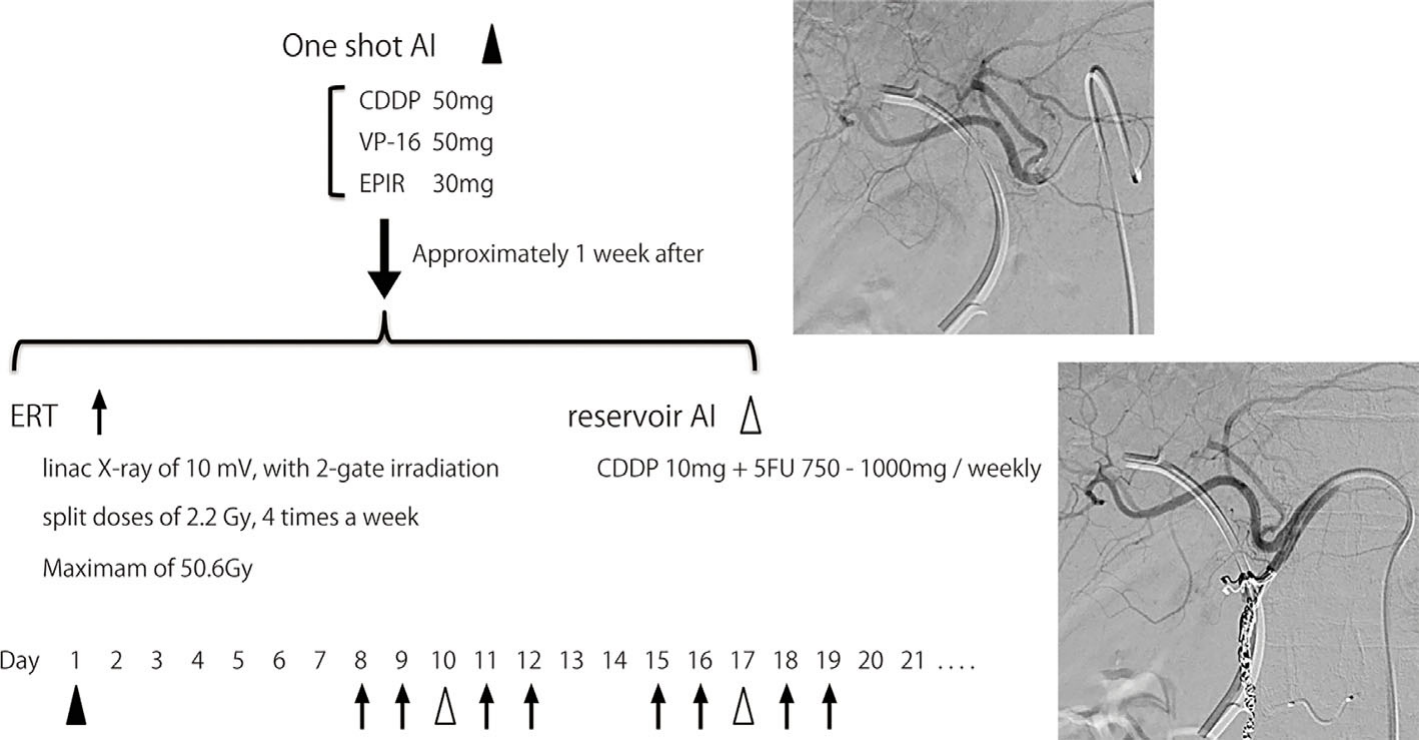
Process of AI+RT combination therapy. One-shot AI consisted of cisplatin 50 mg, etoposide 50 mg and epirubicin 30 mg (EEP therapy). Approximately 1 week after one-shot AI, external beam radiation therapy (ERT) was started. ERT used linac X-ray of 10 mV, with 2-gate irradiation administered to the target as split doses of 2.2 Gy, 4 times a week, with a maximum of 50.6 Gy. The irradiation field includes from the pancreas head to the liver duodenum ligament, focusing on the primary tumor. The reservoir system of AI was embedded in the subcutaneous of the groin area almost at the same time as ERT was started. A catheter was placed by the gastroduodenal artery (GDA) coil method, and the side hole was positioned near the common hepatic artery. AI from the reservoir (reservoir AI) was performed with FP therapy (5FU 750-1,000 mg + cisplatin 10 mg) once a week.

The AI+RT therapy schedule is shown in **Figure 1**. First, when angiography was performed, blood flow modification and one-shot AI was performed. One-shot AI consisted of etoposide 50 mg, epirubicin 30 mg and CDDP 50 mg (EEP therapy). Approximately 1 week after one-shot AI, external beam radiation was started. External radiation used linac X-ray of 10 mV with 2-gate irradiation administered to the target as split doses of 2.2 Gy, 4 times a week, with a maximum of 50.6 Gy. The irradiation field includes from the pancreas head to the liver duodenum ligament, focusing on the primary tumor. The reservoir system of AI was embedded in the subcutaneous of the groin area almost at the same time as external beam radiation was started. A catheter was placed by the gastroduodenal artery (GDA) coil method, and the side hole was positioned near the common hepatic artery. AI from the reservoir (reservoir AI) was performed with FP therapy (5FU 750–1,000 mg + CDDP 10 mg), as is common with AI for liver metastasis (17), once a week. Reservoir AI treatment was ended in cases with disease progression or uncontrolled adverse events. The treatment was continued for about six months and followed by one of the following three policies: 1. Continue AI, 2. Transition to systemic chemotherapy, 3. Follow without treatment. Many patients were transitioning to systemic chemotherapy or follow-up, and reservoir AI was continued after six months in only a few patients whose target lesions were fully covered and controlled by AI.

### Efficacy, Survival, and Safety Assessments

The therapeutic effect of AI+RT was examined based on the response rate (RR), disease control rate (DCR), and adverse events. The objective RR was judged by CT based on the response evaluation criteria in solid tumors version 1.1 (RECIST v1.1). CT was performed before AI+RT and three to six months after the start of the treatment.

Since AI+RT was completed within about six months, even in effective cases, the progression-free survival (PFS) in this study was evaluated from the start of AI+RT to the time of disease progression.

Adverse events were graded according to the Common Terminology Criteria for Adverse Events version 4.0 (CTACE v4.0).

### Statistical Analyses

The following nominal variables were compared between groups using Fisher’s exact test: age, gender, performance status (PS), jaundice, albumin, CA19-9, CEA, lesion site, tumor diameter, UICC T (hepatoduodenal mesentery invasion, arterial invasion, portal vein invasion), UICC N, UICC M (liver metastasis, distant metastasis, peritoneal dissemination), number of AI, 5FU total volume, CDDP total volume, completion of RT, response of AI+RT, and transition to systemic chemotherapy. A logistic regression analysis was used for the multivariate analysis. In the survival analyses, the probability of the overall survival (OS) and PFS were determined by the Kaplan-Meier method with a log-rank test and Cox’s proportional-hazards regression model. Statistical analyses were performed using the Graph Pad PRISM (Version 5.0a; GraphPad Software, Inc., La Jolla, CA, USA), SPSS and R software programs. The level of significance was set at p < 0.05.

## Results

### Patient Background

The patient background characteristics and treatment content are shown in **Table 1**.

**Table 1:** The baseline characteristics and treatment content of the patients and lesions.

Total, n	Bile Tract Cancer (BTC) n = 52
**Age: median ± SD (range)**	70.0 ± 9.1 (40 - 86)
**Gender (male: female)**	25: 27
**Performance status (0:1:2)**	20: 26: 6
**Jaundice (No: Yes)**	23: 29
**Albumin value (ng/ml): median ± SD (range)**	3.5 ± 0.48 (2.3–4.3)
**CEA value (ng/ml): median ± SD (range)**	2.4 ± 59.7 (0.5–413)
**CA19-9 value (U/ml): median ± SD (range)**	81.8 ± 16039 (2–94800)
**Tumor diameter (mm):median ± SD (range)**	33.8 ± 10.4 (13.8–68.6)
**Hepatoduodenal mesentry invasion (No: Yes)**	27: 25
**Arterial invasion (No: Yes)**	35: 17
**Portal vein invasion (No: Yes)**	38: 14
**Lymph node metastasis (No: Yes)**	20: 32
**Liver metastasis (No: Yes)**	49: 13
**Distant metastasis (No: Yes)**	50: 2
**Peritoneal dissemination (No: Yes)**	50: 2
**UICC 8 (2: 3: 4)**	18: 20: 14
**Number of AI: mean ± SD (range)**	13.0 ± 7.9 (3–39)
**5FU total volume (mg): median ± SD (range)**	9,750 ± 6,982 (1,500 – 38,000)
**CDDP total volume (mg): median ± SD (range)**	160.0 ± 76.7 (50–430)
**Completion of RT (No: Yes)**	3: 49
**Transition to systemic chemotherapy (No: Yes)**	24: 28
**Biliary drainage (No: Yes)**	19: 33

AI, intraarterial chemotherapy; CDDP, cisplatin; RT, radiation therapy.

The 52 cases of unresectable BTCs showed no significant differences in characteristics between men and women, and the median age was about 70 years old. Regarding the PS, a PS of 1 was the most frequent, being found in 26 cases, followed by PS0 in 20 cases and PS2 in 6 cases. Blood tests showed jaundice in 29 cases, which was more than half of all BTC cases. The median CA19-9 was high at 81.8 U/ml in all BTC. The maximum CEA value was also high at 413 ng/ml, but 39 cases were within the standard value, so the median CEA value stayed in normal.

Regarding tumor factors, the average tumor diameter was 33.8 mm. Local invasion was hepatoduodenal mesentery in 25 cases, arterial in 17 and portal vein in 14. Regarding metastatic lesions, lymph node metastasis was observed in 32 cases, liver metastasis in 13 cases and distant metastasis and peritoneal dissemination in 2 cases each. Among the UICC stages, stage 3 was slightly more common.

The number of intra-arterial injections was 13, and the median doses of 5FU and CDDP were 9,750 mg and 160 mg, respectively. RT was completed in 49 cases, giving a completion rate of 94.2%. The breakdown of the three discontinued cases was as follows: one case was rejected due to intellectual disability, one case progressed with pleural dissemination suspected before RT, and 1 case discontinued due to gastric ulcer. About half of cases (53.8%) transferred to systemic chemotherapy after AI+RT combination therapy. The second-line therapies after AI+RT were GC in 18 cases, S-1 + CDDP in 4 cases, S-1 in 3 cases, GS in 1case, UFT in 1 case, and surgery in 1 case. Biliary drainage was performed in 33 cases.

### Anti-Tumor Effect of AI+RT

**Table 2** shows the extremely high anti-tumor effect of AI+RT with an RR of 40.4% and DCR of 96.2%. We also deemed two cases in whom recurrence of cancer had not been observed for over five years to have a complete response.

**Table 2:** The summary of overall response.

Total, n	All Bile Tract n = 52
CR: PR: SD:PD	2: 19: 29:2
Response Rate (RR)	40.4% (21/52)
Disease Control Rate (DCR)	96.2% (50/52)

CR, complete response; PR, partial response; SD, stable disease; PD, progression disease.

### The OS, PFS, and Prognostic Factors of AI+RT

**Figure 2:**
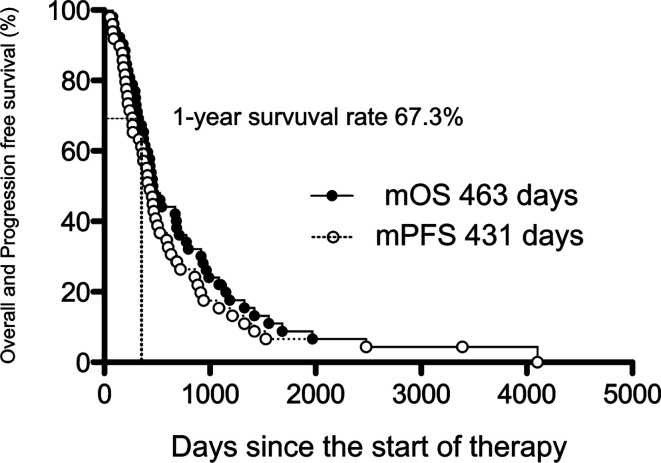
The median overall and progression-free survival of AI+RT in biliary tract cancer (BTC). Kaplan-Meier estimates of the mOS and mPFS. The mOS, mPFS, and 1-year survival rate were 463 and 431 days (15.4 and 14.3 months) and 67.3%, respectively.

The prognosis of BTC was analyzed by the Kaplan-Meier method (**Figure 2**), and the median OS (mOS) was 463 days (15.4 months), and the 1-year survival rate was high at 67.3%. The median PFS (mPFS) from the start of AI+RT until disease progression was 431 days (14.3 months).

**Figure 3:**
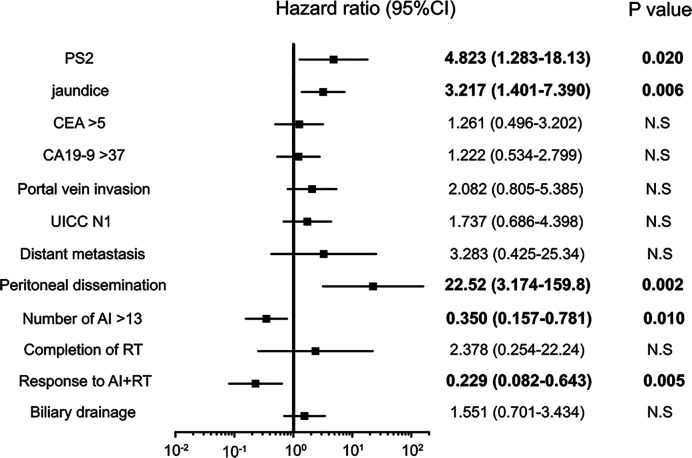
Significant independent prognostic factors of biliary tract cancer (BTC). PS2, jaundice, peritoneal dissemination, number of AI sessions, response to AI+RT and biliary drainage were significant independent prognostic factors of BTC. Peritoneal dissemination had the highest hazard ratio (HR) at 22.5, while a response to AI+RT showed the lowest HR at 0.23.

A univariate analysis was conducted *via* a log rank test (**Tables 3A**, **B**), and a significant difference was noted in 12 items (PS, CA19-9 value, CEA value, jaundice, portal vein invasion, lymph node metastasis, distant metastasis, peritoneal dissemination, number of AI, completion of RT, response to AI+RT, and biliary drainage). Among these, the prognosis was particularly good for the following factors: response to AI+RT (1151 days), no lymph node metastasis (940 days) and no jaundice (915 days). A multivariate analysis using the 12 significant items was performed by proportional hazard model, and PS2 (HR: 4.82, p=0.020), jaundice (HR: 3.22, p=0.005), peritoneal dissemination (HR: 22.5, p=0.002), number of AI sessions (HR: 0.35, p=0.010), and response to AI+RT (HR: 0.23, p=0.005) were identified as significant independent prognostic factors **(****Figure 3****)**.

**Table 3A:** Prognostic factors in all biliary tract cancers (BTCs): univariate analysis (patient and tumor factors).

Prognostic factor		n	Survival time (day)	P value
Age	70 years old more Younger than 70	29 23	430 709	N.S
Gender	Male Female	25 27	444 467	N.S
PS	2 0, 1	6 46	271.5 544	<0.001
Jaundice	Yes No	29 23	371 915	0.002
Albumin	Low (< 3.5) Normal (> 3.5)	28 23	505.5 431	N.S
CEA value	High (> 5) Normal (< 5)	9 39	296 544	0.019
CA19-9 value	High (> 37) Normal (< 37)	31 21	430 775	0.009
Lesion site	GB BD	24 28	535 437.5	N.S
Tumor diameter	> 33.8mm < 33.8mm	26 24	437 505.5	N.S
Hepatoduodenal mesentery invasion	Yes No	25 27	526 453	N.S
Arterial invasion	Yes No	17 35	371 670	N.S
Portal vein invasion	Yes No	14 38	347 544	0.047
Lymph node metastasis	Yes No	32 20	442 940	0.002
Liver metastasis	Yes No	13 39	467 459	N.S
Distant metastasis	Yes No	2 50	198.5 496.5	0.004
Peritoneal dissemination	Yes No	2 50	209 496.5	0.002

PS, performance status.

**Table 3B:** Prognostic factors in all BTCs: univariate analysis (therapy factors).

Prognostic factor		n	Survival time (day)	P value
Number of AI	< 13 > 13	19 30	313 535	0.040
5FU total volume	< 9750mg > 9750mg	24 25	401 526	N.S
CDDP total volume	< 160mg > 160mg	22 27	313 544	0.097
Completion of RT	No Yes	3 49	241 526	0.002
Respose to AI+RT	No Yes	31 21	371 1,151	<0.001
Transition to CT	No Yes	24 28	361.5 670	0.092
Transition to GC	No Yes	34 18	431 506	N.S
Transition to SP	No Yes	48 4	463 629	N.S
Transition to GS	No Yes	51 1	459 990	N.S
Transition to S-1	No Yes	49 3	453 965	N.S
Transition to UFT	No Yes	24 28	459 NA	N.S
Transition to Surgery	No Yes	51 1	459 1,970	N.S
Biliary drainage	No Yes	19 33	795 430	0.038

AI, intraarterial chemotherapy; RT, radiation therapy; CT, systemic chemotherapy; GC, gemcitabine plus cisplatin; SP, S-1 plus cisplatin; GS, gemcitabine plus S-1.

### Adverse Events of AI+RT

Grade 3 and 4 hematologic adverse events were infrequent, with leukopenia occurring in 11.5%, neutropenia in 1.9%, anemia in 15.4%, and thrombocytopenia in 11.5% (**Table 4**). Non-hematologic adverse events of the same grade were anorexia in 3.8%, gastroduodenal ulcer in 25.0% and cholangitis in 23.1%, so ulcer and cholangitis were somewhat frequently observed. There were no treatment-related deaths.

**Table 4:** The summary of adverse events.

Total, n	All Bile Tract n = 52	
	All grade (%)	Grade 3,4 (%)
Hematologic		
Leukopenia	32 (61.5)	6 (11.5)
Neutropenia	18 (34.6)	1 (1.9)
anemia	27 (51.9)	8 (15.4)
Thronbocytopenia	29 (55.8)	6 (11.5)
Renal failure	2 (3.8)	0
Non-hematologic		
Anorexia	17 (32.7)	2 (3.8)
Abdominal pain	21 (40.4)	0
Nausea	15 (28.8)	0
Diarreha	0	0
Gastroduodeneal ulcer	19 (36.5)	13 (25.0)
Cholangitis	12 (23.1)	12 (23.1)
Fatigue	9 (17.3)	0
Rash	3 (5.8)	0
Pancreatitis	1 (1.9)	0
Bile duct bleeding	1 (1.9)	1 (1.9)
Liver abcess	1 (1.9)	1 (1.9)
Catheter trouble	2 (3.8)	2 (3.8)
Biliary fistula	1 (1.9)	1 (1.9)

Hematologic adverse events were relatively frequent for all grades: 32 cases with leukocytopenia (61.5%), 18 cases with neutropenia (34.6%), 27 cases with anemia (51.9%), and 29 cases with thrombocytopenia (55.8%). Nonhematologic adverse events of all grades were also relatively frequent: 17 cases with anorexia (32.7%), 21 with abdominal pain (40.4%), 15 with nausea (28.8%), and 9 with fatigue (17.3%). It may be characteristic that gastroduodenal ulcers not studied by standard treatment were recognized in 19 cases (36.5%). The catheter trouble was “bleeding at the catheter indwelling part” and “catheter occlusion” in one case each.

### The Comparison of GBC and BDC: A Subgroup Analysis

Compared with the BDC group, the GBC group showed a slightly higher prevalence of young people and women, lower rates of jaundice, higher CA19-9 levels, more hepatoduodenal mesentery invasion, liver metastases and UICC4. In addition, the GBC group included more cases transitioning to systemic chemotherapy and fewer cases with biliary drainage than the BDC group (**Supplementary Table 1**).

The RR tended to be better in the GBC group (GBC 45.8%, BDC 35.7%), and the DCR was equally good between the groups (**Supplementary Table 2**).

The respective mOS and mPFS values were 535 and 460 days in the GBC group and 438 and 411 days in the BDC group, showing a slightly better prognosis in the GBC group (**Supplementary Figures 1**, **2**). The one-year survival rate showed no marked difference among between the groups.

**Figure 4:**
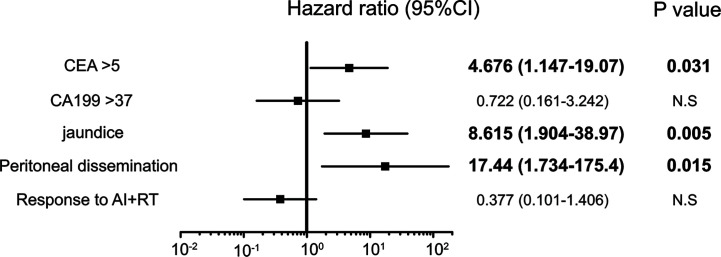
Significant independent prognostic factors of gallbladder cancer (GBC). The CEA value, jaundice and peritoneal dissemination were significant prognostic factors in the multivariate analysis. Peritoneal dissemination had the highest HR at 17.4.

**Figure 5:**
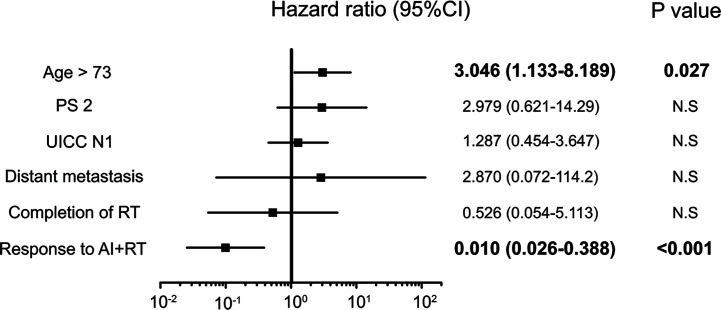
Significant independent prognostic factors of bile duct cancer (BDC). The response to AI+RT and age ≥73 years old were significant prognostic factors in the multivariate analysis. A response to AI+RT had the lowest HR at 0.100.

Five factors were extracted as significant prognostic factors in the GBC group (**Supplementary Tables 3A, B**), and the CEA value (HR: 4.676, p=0.031), jaundice (HR: 8.615, p=0.005), and peritoneal dissemination (HR: 17.44, p = 0.015) were independent prognostic factors in the multivariate analysis with significance **(****Figure 4****)**. Six prognostic factors were extracted in the BDC group (**Supplementary Tables 4A, B**), and a response to AI+RT (HR: 0.100 p<0.001) and age ≥73 years old (HR: 3.046 p=0.027) were significant prognostic factors in the multivariate analysis **(****Figure 5****)**. The incidences of Grades 3 and 4 adverse events showed no significant differences between the groups (**Supplementary Table 5**).

## Discussion

This study examined the usefulness and safety of AI+RT combination therapy for BTC for the first time and proved that the high RR and good mOS and mPFS values.

Previous reports have shown that the RR of systemic chemotherapy, such as GC standard therapy and GS therapy, is still around 20%–30% (7, 8, 11–14); given this, the 40% range of the RR of AI+RT is very high. The mOS of 15.4 months and 1-year survival rate of 67.3% in the AI+RT group seem good compared with the mOS of 11.2 months and 1-year survival rate of 39% for GC therapy in the BT-22 test. In addition, the mPFS of AI+RT was found to be 14.3 months, exceeding 1 year. This good result is considered to be due to the high antitumor effect of six-month AI+RT. Previous reports have found that the mPFS of GC and GS was 5.7–8 months (7, 8, 11, 13), indicating that the mPFS of AI+RT greatly surpassed. This study confirmed that preceding AI+RT in BTCs can delay the time to lesion deterioration.

AI is a method of locally injecting anticancer drugs, targeting the main lesions as well as hepatic infiltration and liver metastasis. It is possible to enhance the effect and reduce adverse events by increasing the local concentration of drug. In addition, its theoretical usefulness in pharmacokinetics has also been reported (18). Thus far, AI chemotherapy has been reported to have a high response rate of 42.9% to 60% (19) and has led to disease control in up to three-quarters of patients with advanced ICC (20, 21). Few reports have mentioned a good prognosis with AI. Only one showed that the three-year survival rate and survival without liver metastases of stage 2 and 3 GBC was better with AI than with systemic chemotherapy (22). Regarding RT, the RR was reported to be high (75% for lymph node lesions of ICC and ≥50% for extrahepatic cholangiocarcinoma) (23, 24), and several early-phase studies have suggested that RT can prolong the survival (23, 25, 26). However, the mOS with RT has been reported to be around one year (24). RT still has issues with its significance in unresectable cases not being clear, as almost all cases have been locally advanced, and phase 3 studies are lacking. Because each modality exerts an antitumor effect in different areas, the combination of AI and RT is therefore expected to achieve a high efficacy in comparison to each monotherapy.

One persistent problem is about AI+RT’s adaptation that it has to be carefully judged because no prospective study has yet been conducted. Among the locally advanced cases, hepatoduodenal mesentery invasion has been reported to be a significantly poor prognostic factor, even in resected cases, with a mOS of around eight months (27). The hepatoduodenal mesentery is abundant in nerve plexus and a lymphatic network that promote the progression of cancer, so mesenteric infiltration has been thought to contribute to a poor prognosis. The mOS of hepatoduodenal mesentery invasion with AI+RT in this study was 14.4 months, which was superior to previously reported surgical cases (27). Both arterial and portal invasion cases tended to have a poor prognosis, but the mOS was relatively good at around 12 months. Therefore, locally advanced cases were considered to be indicated for AI+RT. Regarding metastasis, the outcomes differed between lymph node and liver metastases and distant metastasis and peritoneal dissemination. Lymph node metastasis was a significant prognostic factor in the univariate analysis but not the multivariate analysis, showing an mOS of ≥14 months. In addition, lymph node metastasis is also included in the therapeutic range of this therapy. Cases of hepatic metastasis were reconfirmed to be a good indication for AI+RT, as not only was liver failure due to hepatic metastasis previously reported to be decreased by AI (16), but the liver metastasis cases also had a good mOS of 15.6 months. In contrast, the prognosis was extremely poor in patients with distant metastases and peritoneal dissemination, which existed outside the therapeutic area of AI+RT. The mOS of about 200 days with these metastases did not clearly exceed the prognosis of systemic chemotherapy. Based on the above, the indication of AI+RT was thought to be cases without distant metastasis or peritoneal dissemination.

The multivariate analysis of prognostic factors confirmed that patient factors associated with a poor prognosis were PS2 and jaundice in all BTC, jaundice in GBC, and cases ≥73 years old in BDC. Regarding the treatment, two factors were extracted as significant prognostic factors—complete or partial response with AI+RT and AI ≥13 times—so the response and number of times this therapy was applied were inferred to affect the prognosis. Since BTC, GBC, and BDC had differing prognostic factors, a further analysis is necessary to clarify the indication of AI+RT at each site.

The rate of hematological adverse events of Grades 3 and 4 in AI+RT was about 15%, with neutropenia only observed in about 2% of cases. The BT-22 study observed the following Grade 3 and 4 hematological adverse events: leukopenia in 29.3%, anemia in 36.6%, thrombocytopenia in 39% and neutropenia in 56.1% (8). This study found AI+RT to have less hematologic toxicity than systemic chemotherapy, an outcome attributed to the anticancer drug having little effect on the whole body because it was administered at a high concentration mainly to the primary tumor and its surroundings. However, gastroduodenal ulcers and cholangitis were frequent with AI+RT. Cholangitis was found in 8% to 38% of patients in the BT-22 study (28), but it was not a common adverse event of AI+RT. Ulcers may be unique to AI+RT, as the effect is concentrated locally.

Several limitations associated with the present study warrant mention, including its retrospective nature and single-center setting. In addition, the indications for this therapy were unclear, but the results suggested that cases with peritoneal dissemination and/or distant metastases were not indicative. The influences of age and PS on the efficacy of AI+RT and frequencies of adverse events in each site need to be verified in a prospective study. Furthermore, we were unable to study some molecular factors related to the response rate and survival. Next-generation sequencing should be considered to identify molecular markers for the prediction of efficacy in a future prospective study.

## Conclusion

In summary, AI+RT combination therapy in BTC contributed to a high response rate and long survival. Advances in multidisciplinary treatment, including not only systemic chemotherapy but also AI and RT, are important for improving the prognosis of BTC. We would like to next report the findings of a prospective verification study based on these results.

## Data Availability Statement

The raw data supporting the conclusions of this article will be made available by the authors, without undue reservation.

## Ethics Statement

The studies involving human participants were reviewed and approved by the Medical Ethics Committee of Asahikawa Medical University the Medical Ethics Committee of Asahikawa Kousei Hospital. Written informed consent for participation was not required for this study in accordance with the national legislation and the institutional requirements.

## Author Contributions

Conception and design: TG, HSai. Provision of study material or patients: TG, HSai, TK, AF, NY. Enforce of inspection and treatment: TG, HSai, TK, AF, NY, KH, AT. Collection and assembly of data: TG, TK, TU, SF, HSat. Data analysis and interpretation: TG, JS. Manuscript writing: TG, JS. Provided valuable opinions in manuscript: HSai, NY, MF. Critical revision of the manuscript: HSai, MF. All authors contributed to the article and approved the submitted version.

## Conflict of Interest

Author MF received grants and personal fees from Yakult Honsha Co. Ltd., Nippon Kayaku Co. Ltd. and Pfizer Inc.The remaining authors declare that the research was conducted in the absence of any commercial or financial relationships that could be construed as a potential conflict of interest.

## Supplementary Material

Supplementary Figure 1 The median OS of GBC and BDC. Kaplan-Meier estimates of the mOS of GBC and BDC. The mOS of GBC was 535 days, and that of BDC was 438 days, showing a slightly better tendency with GBC

Supplementary Figure 2 The median PFS of GBC and BDC. Kaplan-Meier estimates of the mPFS of GBC and BDC. The mPFS of GBC was 460 days, and that of BDC was 411 days.

## Abbreviations

AI, intraarterial chemotherapy; AI+RT, intraarterial chemotherapy plus radiation therapy; BDC, bile duct cancer; BTC, biliary tract cancer; CDDP, cisplatin; CapeOX, capecitabine plus oxaliplatin; DCR, disease control rate; EHC, extrahepatic cholangiocarcinoma; EUS, endoscopic ultrasound; GBC, gallbladder cancer; GC, gemcitabine and cisplatin; GDA, gastroduodenal artery; GEMOX, gemcitabine plus oxaliplatin; GS, gemcitabine plus S-1; HR, hazard ratio; ICC, intrahepatic cholangiocarcinoma; OS, overall survival; PFS, progression-free survival; PS, performance status; reservoir AI, intraarterial chemotherapy from the reservoir; RR, response rate; RT, radiation therapy; SP, S-1 plus cisplatin.
