# Supplementary figures and images for: High Response Rate and Prolonged Survival of Unresectable Biliary Tract Cancer Treated With a New Combination Therapy Consisting of Intraarterial Chemotherapy Plus Radiotherapy
Source: Front Oncol. 2020 Nov 17;10:597813. doi: 10.3389/fonc.2020.597813 (PMC7707151; doi:10.3389/fonc.2020.597813)

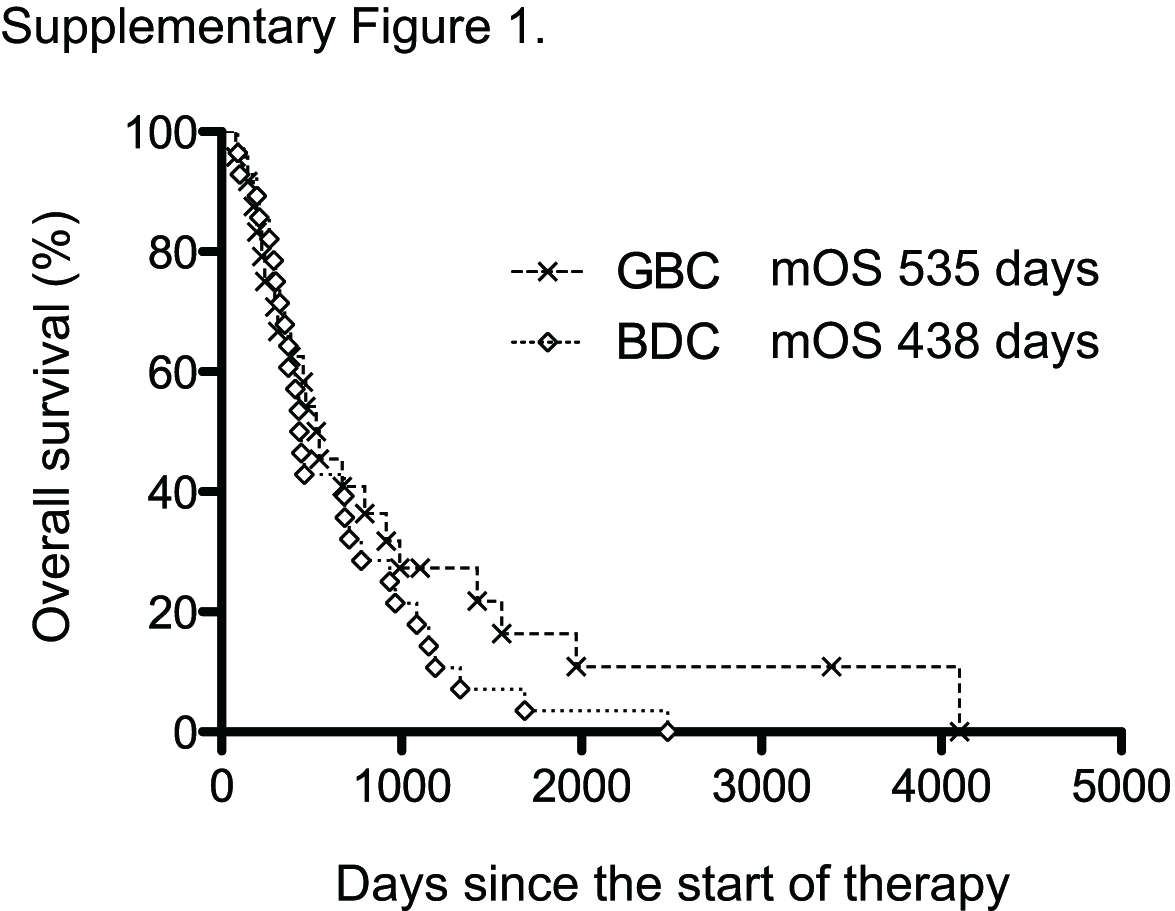

Supplement: Supplementary Figure 1 — The median OS of GBC and BDC. Kaplan-Meier estimates of the mOS of GBC and BDC. The mOS of GBC was 535 days, and that of BDC was 438 days, showing a slightly better tendency with GBC [file Image_1.tif]

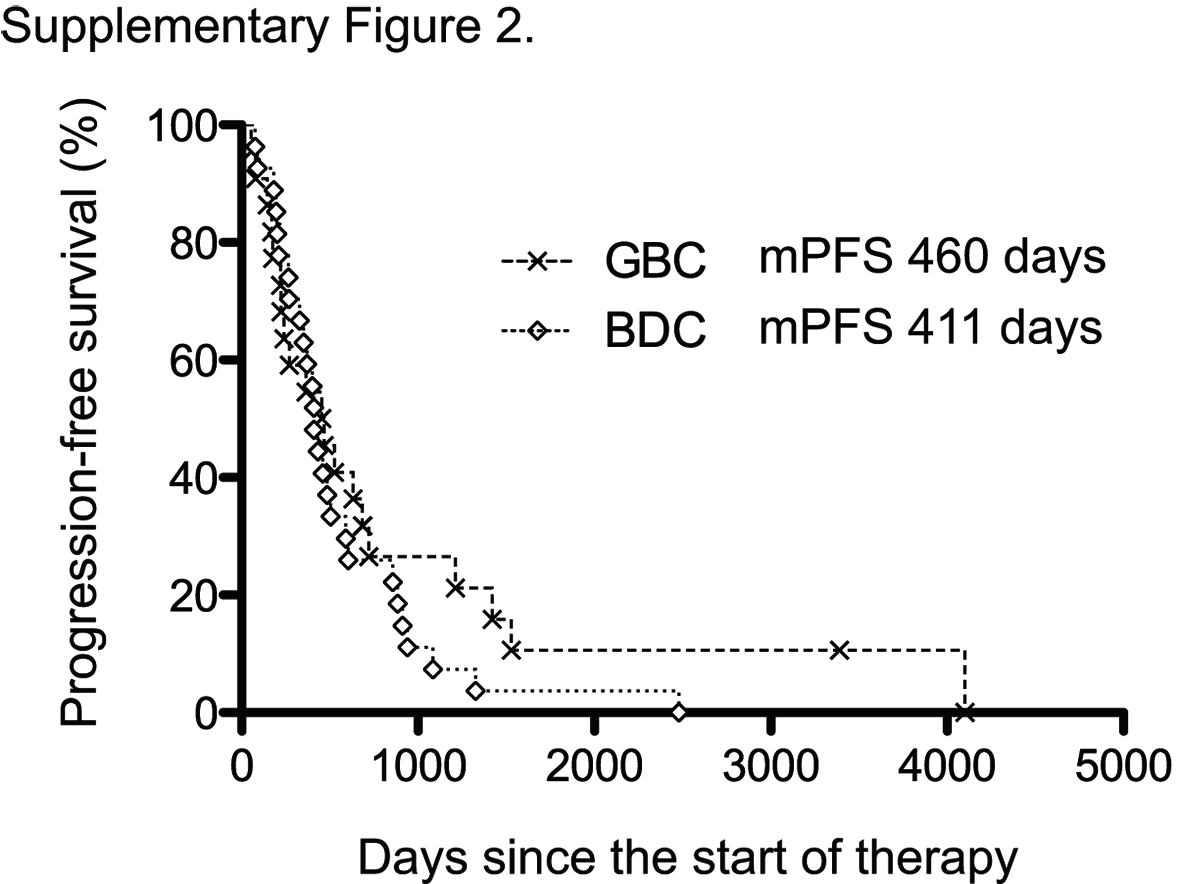

Supplement: Supplementary Figure 2 — The median PFS of GBC and BDC. Kaplan-Meier estimates of the mPFS of GBC and BDC. The mPFS of GBC was 460 days, and that of BDC was 411 days. [file Image_2.tif]
