# Supplementary material for: High Response Rate and Prolonged Survival of Unresectable Biliary Tract Cancer Treated With a New Combination Therapy Consisting of Intraarterial Chemotherapy Plus Radiotherapy
Source: Front Oncol. 2020 Nov 17;10:597813. doi: 10.3389/fonc.2020.597813 (PMC7707151; doi:10.3389/fonc.2020.597813)
Supplement: Supplementary file 3 [file Table_1.docx]

| **Total, n** | **Gallbladder Cancer**  **(GBC)**  **n = 24** | **Bile Duct Cancer**  **(BDC)**  **n = 28** |
| --- | --- | --- |
| **Age: median ± SD (range)** | 65.0 ± 10.0 (40 - 77) | 73.0 ± 5.8 (61 - 86) |
| **Gender (male : female)** | 9 : 15 | 16 : 12 |
| **Performance status (0:1:2)** | 11: 11 :2 | 9 : 15 : 4 |
| **Jaundice (No : Yes)** | 17 : 7 | 6 : 22 |
| **Albumin value (ng/ml): median ± SD (range)** | 3.5 ± 0.28 (2.9 – 4.3) | 3.4 ± 0.57 (2.3 – 4.1) |
| **CEA value (ng/ml): median ± SD (range)** | 2.5 ± 84.3 (0.5 – 413) | 2.4 ± 2.1 (1.1 – 10.1) |
| **CA19-9 value (U/ml): median ± SD (range)** | 657.4 ± 22436 (4.8 – 94800) | 57.5 ± 341.5 (2 – 1355.2) |
| **Tumor diameter (mm):median ± SD (range)** | 37.1 ± 12.4 (13.8 – 68.6) | 32.1 ± 7.5 (17.4 – 52.5) |
| **Hepatoduodenal mesentry invasion (No : Yes)** | 9 : 15 | 18 : 10 |
| **Arterial invasion (No : Yes)** | 18 : 6 | 17 : 11 |
| **Portal vein invasion (No : Yes)** | 18 : 6 | 20 : 8 |
| **Lymph node metastasis (No : Yes)** | 10 : 14 | 10 : 18 |
| **Liver metastasis (No : Yes)** | 12 : 12 | 27 : 1 |
| **Distant metastasis (No : Yes)** | 23 : 1 | 27 : 1 |
| **Peritoneal dissemination (No : Yes)** | 22 : 2 | 28 : 0 |
| **UICC 8 (2 : 3: 4)** | 2 : 10 : 12 | 16 : 10 : 2 |
| **Number of AI: mean ± SD (range)** | 13.0 ± 8.4 (3 – 35) | 13.5 ± 7.5 (4 – 39) |
| **5FU total volume (mg): median ± SD (range)** | 9750 ± 6441 (1500 – 26250) | 9250 ± 7399 (2250 – 38000) |
| **CDDP total volume (mg): median ± SD (range)** | 170.0 ± 78.1 (50 – 360) | 160.0 ± 75.2 (60 – 430) |
| **Completion of RT (No : Yes)** | 1 : 23 | 2 : 26 |
| **Transition to systemic chemotherapy (No : Yes)** | 8 : 16 | 16 : 12 |
| **Biliary drainage (No : Yes)** | 16 : 8 | 3 : 25 |

**Supplementary Table 1. The baseline characteristics and treatment content of the patients and lesions**

AI, intraarterial chemotherapy; CDDP, cisplatin; RT, radiation therapy.
