# Supplementary material for: High Response Rate and Prolonged Survival of Unresectable Biliary Tract Cancer Treated With a New Combination Therapy Consisting of Intraarterial Chemotherapy Plus Radiotherapy
Source: Front Oncol. 2020 Nov 17;10:597813. doi: 10.3389/fonc.2020.597813 (PMC7707151; doi:10.3389/fonc.2020.597813)
Supplement: Supplementary file 4 [file Table_2.docx]

| **Total, n** | Gallbladder  n = 24 | Bile duct  n = 28 |
| --- | --- | --- |
| **CR : PR : SD :PD** | 1 : 10 : 12: 1 | 1: 9 : 17 : 1 |
| **Response Rate**  **(RR)** | **45.8 %**  (11 / 24) | **35.7 %**  (10 / 28) |
| **Disease Control Rate (DCR)** | **95.8 %**  (23 / 24) | **96.4 %**  (27 /28) |

**Table 2. The summary of overall response**

CR, complete response; PR, partial response; SD, stable disease; PD, progression disease.
