# Supplementary material for: High Response Rate and Prolonged Survival of Unresectable Biliary Tract Cancer Treated With a New Combination Therapy Consisting of Intraarterial Chemotherapy Plus Radiotherapy
Source: Front Oncol. 2020 Nov 17;10:597813. doi: 10.3389/fonc.2020.597813 (PMC7707151; doi:10.3389/fonc.2020.597813)
Supplement: Supplementary file 5 [file Table_3.docx]

| **Prognostic factor** | | **n** | **Survival time (day)** | **P value** |
| --- | --- | --- | --- | --- |
| **Age** | 65 years old more  Younger than 65 | 13  11 | 670  313 | N.S |
| **Gender** | Male  Female | 9  15 | 670  526 | N.S |
| **PS** | 2  0, 1 | 2  22 | 347  544 | N.S |
| **Jaundice** | Yes  No | 7  17 | 241  **915** | **<0.001** |
| **Albumin** | Low (< 3.5)  Normal (> 3.5) | 8  15 | 419.5  544 | N.S |
| **CEA value** | High (> 5)  Normal (< 5) | 7  16 | 241  **795** | **0.002** |
| **CA19-9 value** | High (> 37)  Normal (< 37) | 16  8 | 418.5  **1108.5** | **0.049** |
| **Tumor diameter** | > 37.1mm  < 37.1mm | 11  11 | 670  384 | N.S |
| **Hepatoduodenal mesentery invasion** | Yes  No | 15  9 | 526  670 | N.S |
| **Arterial invasion** | Yes  No | 6  18 | 605.5  535 | N.S |
| **Portal vein invasion** | Yes  No | 6  18 | 304.5  544 | N.S |
| **Lymph node metastasis** | Yes  No | 14  10 | 460  1108.5 | 0.056 |
| **Liver metastasis** | Yes  No | 12  12 | 460  669.5 | N.S |
| **Distant metastasis** | Yes  No | 1  23 | 296  544 | N.S |
| **Peritoneal dissemination** | Yes  No | 2  22 | 209  **544** | **0.009** |

**Supplementary Table 3a. Prognostic factors in GBCs: univariate analysis (patient and tumor factors)**

PS, performance status.

| **Prognostic factor** | | **n** | **Survival time (day)** | **P value** |
| --- | --- | --- | --- | --- |
| **Number of AI** | < 13  > 13 | 10  13 | 304.5  670 | 0.085 |
| **5FU total volume** | < 9750mg  > 9750mg | 10  13 | 381.5  544 | N.S |
| **CDDP total volume** | < 170mg  > 170mg | 11  12 | 313  607 | N.S |
| **Completion of RT** | No  Yes | 1  23 | 241  544 | N.S |
| **Respose to AI+RT** | No  Yes | 13  11 | 453  **1422** | **0.018** |
| **Transition to CT** | No  Yes | 8  16 | 232  544 | N.S |
| **Biliary drainage** | No  Yes | 16  8 | 544  269 | 0.095 |

**Supplementary Table 3b. Prognostic factors in GBCs: univariate analysis (therapy factors)**

AI, intraarterial chemotherapy; RT, radiation therapy; CT, systemic chemotherapy.
