# Supplementary material for: High Response Rate and Prolonged Survival of Unresectable Biliary Tract Cancer Treated With a New Combination Therapy Consisting of Intraarterial Chemotherapy Plus Radiotherapy
Source: Front Oncol. 2020 Nov 17;10:597813. doi: 10.3389/fonc.2020.597813 (PMC7707151; doi:10.3389/fonc.2020.597813)
Supplement: Supplementary file 6 [file Table_4.docx]

| **Prognostic factor** | | **N** | **Survival time (day)** | **P value** |
| --- | --- | --- | --- | --- |
| **Age** | 73 years old more  Younger than 73 | 15  13 | 352  **709** | **0.029** |
| **Gender** | Male  Female | 16  12 | 437.5  444.5 | N.S |
| **PS** | 2  0, 1 | 4  24 | 201.5  **571** | **<0.001** |
| **Jaundice** | Yes  No | 22  6 | 437  698 | N.S |
| **Albumin** | Low (< 3.5)  Normal (> 3.5) | 15  13 | 431  459 | N.S |
| **CEA value** | High (> 5)  Normal (< 5) | 2  23 | 684  430 | N.S |
| **CA19-9 value** | High (> 37)  Normal (< 37) | 15  13 | 430  709 | N.S |
| **Tumor diameter** | > 32.1mm  < 32.1mm | 14  14 | 445  437 | N.S |
| **Hepatoduodenal mesentery invasion** | Yes  No | 10  18 | 571  420 | N.S |
| **Arterial invasion** | Yes  No | 11  17 | 371  685 | N.S |
| **Portal vein invasion** | Yes  No | 8  20 | 400.5  571 | N.S |
| **Lymph node metastasis** | Yes  No | 18  10 | 430.5  **764.5** | **0.024** |
| **Liver metastasis** | Yes  No | 1  27 | 685  431 | N.S |
| **Distant metastasis** | Yes  No | 1  27 | 101  **444** | **<0.001** |
| **Peritoneal dissemination** | Yes  No | 0  28 | -  438 | N.S |

**Supplementary Table 4a. Prognostic factors in BDCs: univariate analysis (patient and tumor factors)**

PS, performance status.

**Supplementary Table 4b. Prognostic factors in BDCs: univariate analysis (therapy factors)**

AI, intraarterial chemotherapy; RT, radiation therapy; CT, systemic chemotherapy.

| **Prognostic factor** | | **n** | **Survival time (day)** | **P value** |
| --- | --- | --- | --- | --- |
| **Number of AI** | < 13  > 13 | 9  17 | 323  444 | N.S |
| **5FU total volume** | < 9250mg  > 9250mg | 13  13 | 431  430 | N.S |
| **CDDP total volume** | < 160mg  > 160mg | 12  14 | 312.5  563.5 | 0.052 |
| **Completion of RT** | No  Yes | 2  26 | 212  **451.5** | **0.010** |
| **Respose to AI+RT** | No  Yes | 18  10 | 370.5  **1118** | **<0.001** |
| **Transition to CT** | No  Yes | 16  12 | 390.5  855 | N.S |
| **Biliary drainage** | No  Yes | 3  25 | 935  431 | N.S |
