# Supplementary material for: High Response Rate and Prolonged Survival of Unresectable Biliary Tract Cancer Treated With a New Combination Therapy Consisting of Intraarterial Chemotherapy Plus Radiotherapy
Source: Front Oncol. 2020 Nov 17;10:597813. doi: 10.3389/fonc.2020.597813 (PMC7707151; doi:10.3389/fonc.2020.597813)
Supplement: Supplementary file 7 [file Table_5.docx]

| **Total, n** | **Gallbladder**  **n = 24** | | **Bile duct**  **n = 28** | |
| --- | --- | --- | --- | --- |
|  | **All grade (%)** | **Grade 3,4 (%)** | **All grade (%)** | **Grade 3,4 (%)** |
| **Hematologic**  **Leukopenia**  **Neutropenia**  **anemia**  **Thronbocytopenia**  **Renal failure** | 17 (70.8)  9 (37.8)  13 (54.2)  12 (50.0)  0 | 2 (8.3)  0  4 (16.7)  4 (16.7)  0 | 15 (53.6)  9 (32.1)  14 (50.0)  17 (60.7)  2 (7.1) | 4 (14.3)  1 (3.6)  4 (14.3)  2 (7.1)  0 |
| **Non-hematologic**  **Anorexia**  **Abdominal pain**  **Nausea**  **Diarreha**  **Gastroduodeneal ulcer**  **Cholangitis**  **Fatigue**  **Rash**  **Pancreatitis**  **Bile duct bleeding**  **Liver abcess**  **Catheter trouble**  **Biliary fistula** | 10 (41.7)  12 (50.0)  10 (41.7)  0  12 (50.0)  7 (29.2)  5 (20.8)  1 (4.2)  0  1 (4.2)  0  1 (4.2)  0 | 0  0  0  0  7 (29.2)  7 (29.2)  0  0  0  1 (4.2)  0  1 (4.2)  0 | 7 (25.0)  9 (32.1)  5 (17.9)  0  7 (25.0)  5 (17.9)  4 (14.3)  2 (7.1)  1 (3.6)  0  1 (3.6)  1 (3.6)  1 (3.6) | 2 (7.1)  0  0  0  6 (21.4)  5 (17.9)  0  0  0  0  1 (3.6)  1 (3.6)  1 (3.6) |

**Supplementary Table 5. The summary of adverse events**
